# Enhancement of β-Alanine Biosynthesis in *Escherichia coli* Based on Multivariate Modular Metabolic Engineering

**DOI:** 10.3390/biology10101017

**Published:** 2021-10-09

**Authors:** Jian Xu, Li Zhou, Zhemin Zhou

**Affiliations:** The Key Laboratory of Industrial Biotechnology of Ministry of Education, School of Biotechnology, Jiangnan University, Wuxi 214122, China; xujiane_mail@163.com (J.X.); Lizhou@jiangnan.edu.cn (L.Z.)

**Keywords:** metabolic engineering, synthetic biology, multivariate modular metabolic engineering, pathway balance

## Abstract

**Simple Summary:**

Pathway balancing is a common and critical challenge for the construction of microbial cell factories using metabolic engineering approaches. However, semi-rational or non-rational manipulation might lead to metabolic imbalances, which may further impact pathway efficiency, and ultimately impact the growth performance and production performance of a microbial cell factory. In this study, the multivariate modular metabolic engineering was employed to engineer the β-alanine biosynthesis pathway and keep the balance of metabolic flux among the whole metabolic network, rationally and systematically. Ultimately, 37.9 g/L β-alanine was generated in fed-batch fermentation. Novel strategies reported in this study were meaningful to the application and diffusion in β-alanine industrial production.

**Abstract:**

β-alanine is widely used as an intermediate in industrial production. However, the low production of microbial cell factories limits its further application. Here, to improve the biosynthesis production of β-alanine in *Escherichia coli*, multivariate modular metabolic engineering was recruited to manipulate the β-alanine biosynthesis pathway through keeping the balance of metabolic flux among the whole metabolic network. The β-alanine biosynthesis pathway was separated into three modules: the β-alanine biosynthesis module, TCA module, and glycolysis module. Global regulation was performed throughout the entire β-alanine biosynthesis pathway rationally and systematically by optimizing metabolic flux, overcoming metabolic bottlenecks and weakening branch pathways. As a result, metabolic flux was channeled in the direction of β-alanine biosynthesis without huge metabolic burden, and 37.9 g/L β-alanine was generated by engineered *Escherichia coli* strain B0016-07 in fed-batch fermentation. This study was meaningful to the synthetic biology of β-alanine industrial production.

## 1. Introduction

β-alanine, which is the sole type of natural β-amino acid, is widely used as an intermediate in industrial production, such as the manufacture of nanocomposites [1], formaldehyde scavengers [2], and acrylamide and acrylonitrile [3]. However, the industrial process currently used for β-alanine production relies on the reaction of acrylic acid and ammonium carbonates under high-temperature and -pressure conditions [4], being highly dependent on petrochemical resources. Increasing concerns about the depletion of natural resources and environmental pollution have pushed researchers to find alternative technologies on environmentally friendly and sustainable methods for β-alanine production.

The advantages of synthetic biology, such as mild reactions, economical materials, and sustainable processes, have greatly facilitated the development of microbial biosynthesis. Large amounts of high-value-added products have been successfully produced by engineered microbes. As for β-alanine biosynthesis, two approaches belonging to synthetic biology are mainly applied: whole-cell biocatalysts [5] and metabolic engineering [6]. The method of whole-cell biocatalysts emphasizes the reaction from L-aspartate to β-alanine, so this method relies on the addition of L-aspartate, which leads to a higher cost and higher yield [5]. The technique of metabolic engineering can convert inexpensive substrate, such as glucose and glycerol, to β-alanine, so this method can reduce the production cost but the yield is lower [6]. As the method of metabolic engineering is more economical and environmentally friendly, studies should be carried out to optimize this method to increase β-alanine yield.

The construction of excellent microbial cell factories is the basic premise of metabolic engineering approaches, and *Escherichia coli* (*E. coli*) is one of the most excellent chassis for industrial purposes due to its rapid growth, well-known metabolism, and easy genetic manipulation [7,8]. However, semi-rational or non-rational manipulation might lead to metabolic imbalances, which may further impact cellular growth and pathway efficiency [9]. To alleviate the negative influences caused by pathway imbalances, multivariate modular metabolic engineering (MME), which redefined the overall pathway as a collection of distinct modules of the tinker pathway [10,11], is widely applied to enhance the balance in each module and between modules; thus, the performance of the engineered strains is improved [12]. As reported, MME was applied to optimize the expression of several dozen proteins in a complex vitamin B12 biosynthetic pathway to maintain the balance of the metabolic network, and the vitamin B12 yield was increased by more than 250-fold [13]. Moreover, MME was also employed to balance the non-linear pathway and overcome the critical and common challenge of pathway balance in rosmarinic acid biosynthesis, and the rosmarinic acid yield was increased by 38-fold [14]. Notably, with the application of MME, sophisticated and interrelated networks can be regulated rationally and systematically to maintain the metabolic flux balance in vivo.

Three candidate biosynthesis pathways that favor the accumulation of oxaloacetate/fumarate, which are the precursors of β-alanine biosynthesis, have been summarized and reported: through the oxidative branch of the tricarboxylic acid (TCA) cycle, through the glyoxylate cycle, and through the reductive branch of the TCA cycle [15]. The first two routes are oxidation reactions and are engaged in the aerobic β-alanine biosynthesis pathway. The third is a reduction reaction, which is involved in the anaerobic β-alanine biosynthesis pathway. Studies have been carried out to produce β-alanine via the first two metabolic pathways, since β-alanine production can be generated more efficiently in aerobic fermentation compared with anaerobic fermentation. As the chassis was manipulated round by round, 32.3 g/L β-alanine was generated [16]. However, pleiotropic effects, such as pathway imbalance and metabolic burden, were generated by unsystematic manipulation, leading to a decrease in biomass and β-alanine production [16,17].

In this study, *E. coli* was rationally and systematically engineered for the production of β-alanine. The MME strategy was applied to channel the metabolic flux in the direction of β-alanine biosynthesis with the aim of maintaining the balance of the intracellular metabolic network. The β-alanine biosynthesis pathway was separated into three modules: the β-alanine biosynthesis module, TCA module, and glycolysis module. Global regulation was performed throughout the entire β-alanine biosynthesis pathway rationally and systematically; as a result, 37.9 g/L β-alanine was generated in fed-batch fermentation.

## 2. Materials and Methods

### 2.1. Strains and Plasmids

The genotypes of the *E. coli* used in this study are listed in Table 1. *E. coli* strain B0016-082BB was previously constructed for β-alanine generation through the knockout of bypass and competitive pathways and the overexpression of the *panD* gene from *Corynebacterium glutamicum* (Cg*panD*). The red recombination strategy was used for the engineering of the microbial cell factories. Plasmids were constructed based on the chassis of pET24a and pCDF. The gene of *lacI* on both plasmids was deleted and the T7 promoter on both plasmids was replaced with a *p_L_* promoter. The antibiotic resistance gene of pCDF was replaced with an ampicillin resistance gene (*ampr*). The modified pET24a and pCDF were renamed pETPL and pCDFPL, respectively. Heterologous genes were codon optimized based on the *E. coli* codon preference and cloned into plasmids using the ClonExpress II One Step Cloning Kit (Vazyme Biotech, Nanjing, China). The primers used in this study are listed in Table S1.

### 2.2. Media and Culture Conditions

Luria–Bertani (LB) medium was recruited for plasmid construction, gene manipulation, and active culture. Seed cultures were prepared in 50 mL of LB medium and incubated in a rotary shaker (ZQZY-C8V, Zhichu, Shanghai, China) with shaking at 200 rpm at 37 °C overnight, and were subsequently centrifuged at 4 °C and suspended in M9Y medium to form a seed suspension. Flasks with M9Y medium were incubated with shaking at 200 rpm and 37 °C, as the seed suspension was inoculated into the M9Y medium to reach an initial *OD*_600_ of 0.05. A volume of 500 μL of sterile glycerol (50%, *w/v*) was fed four times per day periodically, and the pH was regulated at 7 with the addition of 100 g/L NaHCO_3_. Experiments were performed in triplicate.

The fed-batch fermentation was performed in 2 L of M9Y medium contained in a 5 L bioreactor (Winpact, Major Science, Saratoga, CA, USA) at 37 °C. The dissolved oxygen was regulated above 45% by adjusting the air flow from 2 to 10 L/min, and the agitation speed, from 200 to 900 rpm. The feed medium was added automatically according to the growth rate of *E. coli* (μ, h^−1^) and the consumption rate of the substrate (qGly, h^−1^) [19].

M9Y medium contained (per L) 1 g of NH_4_Cl, 0.5 g of NaCl, 2 g of yeast extract, 3 g of KH_2_PO_4_, 5 g of glycerol, 5 mL of a metal solution [20], 6 g of Na_2_HPO_4_, and 13.21 g of (NH_4_)_2_SO_4_.

The feed medium contained (per L) 3.67 g of MgSO_4_, 4 g of yeast extract, 4 g of tryptone, 100 g of (NH_4_)_2_SO_4_, and 650 g of glycerol.

### 2.3. Measurement of Amino Acid Concentration

The concentration of amino acids was analyzed using high-performance liquid chromatography equipped with a C18 column (4.6 × 250 mm, Waters, Milford, MA, USA) by using the o-phthaldialdehyde (opa) derivatization method [21,22].

## 3. Results and Discussion

### 3.1. Module Separation of β-Alanine Biosynthesis

**Figure 1:**
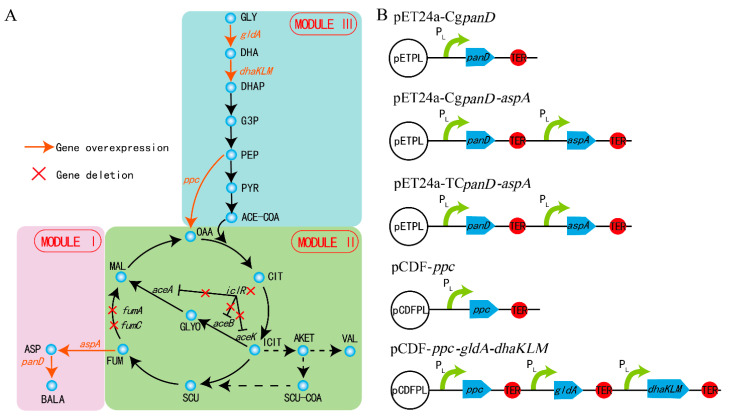
The biosynthesis pathway of β-alanine. The β-alanine biosynthetic pathway was divided into three modules (**A**) and the key genes were overexpressed on the plasmids (**B**). Gly: glycerol; DHA: dihydroxyacetone; DHAP: dihydroxyacetone phosphate; G3P: glyceraldehyde-3-P; PEP: phosphoenolpyruvate; PYR: pyruvate; ACE-COA: acetyl-CoA; OAA: oxaloacetate; CIT: citrate; ICIT: isocitrate; AKET: α- ketoglutarate; VAL: valine; SCU-COA: succinyl-CoA; SUC: succinate; FUM: fumarate; MAL: malate; ASP: L-aspartate; BALA: β-alanine.

The β-alanine biosynthesis metabolic pathway is a long linear pathway, related to the central carbon cycle of the TCA. Irrational genetic manipulation may lead to subtle and far-ranging effects, such as slow cell growth and low metabolic ability [9,16]. To channel the metabolic flux in the direction of β-alanine biosynthesis without pathway imbalance and heavy metabolic burden, the strategy of MME was applied to regulate the β-alanine biosynthesis. The whole metabolic pathway was divided into three modules according to the location and function of every gene within the β-alanine biosynthesis pathway: β-alanine biosynthesis module (module I), TCA module (module II), and glycolysis module (module III) (Figure 1a). Global regulation was performed rationally and systematically using MME throughout the entire β-alanine biosynthesis pathway.

### 3.2. Regulation in the β-Alanine Biosynthesis Module

**Figure 2:**
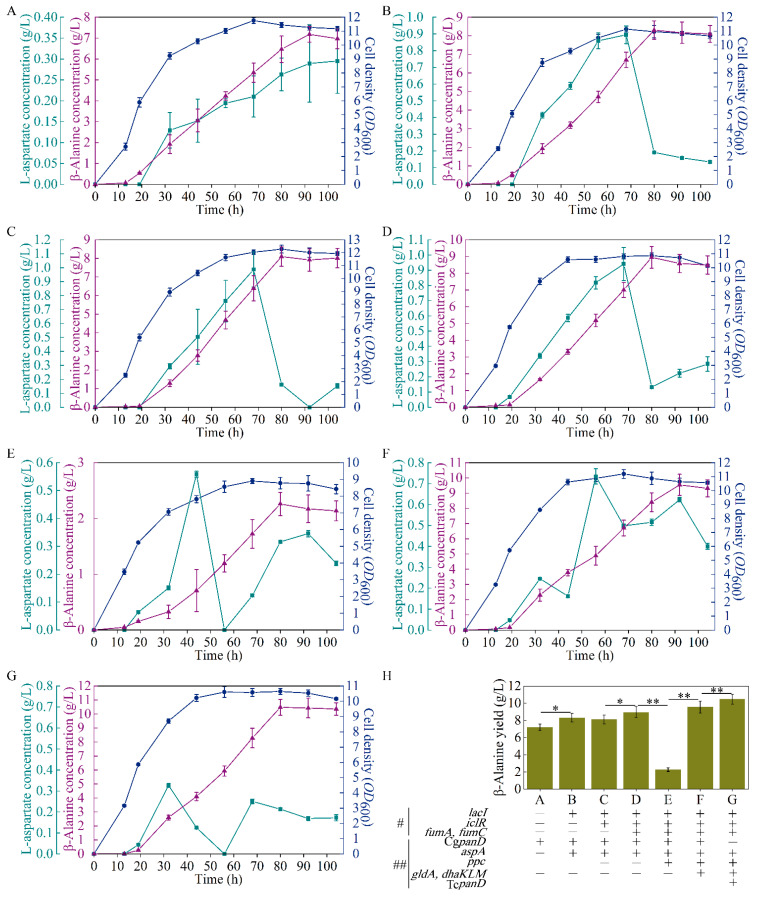
Performance of the engineered strains B0016-01 (**A**), B0016-02 (**B**), B0016-03 (**C**), B0016-04 (**D**), B0016-05 (**E**), B0016-06 (**F**), and B0016-07 (**G**), and comparison of experimental group (**H**) in fermentation. Symbols: cell density, blue circles; β-alanine concentration, purple triangles; L-aspartate concentration, green squares; gene(s) deletion, #; gene(s) overexpression, ##; *p* < 0.05, *; *p* < 0.01, **.

As reported, the reaction from L-aspartate to β-alanine, catalyzed by L-aspartate-α-decarboxylase, is the rate-limiting step of β-alanine generation, and this bottleneck can be enhanced with the overexpression of the *panD* gene [17]. A *E. coli* strain B0016-082BB was previously obtained for β-alanine generation through the deletion of bypass pathways and genome insertion Cg*panD*, with about 1 g/L of β-alanine achieved (13). To further overcome this rate-limiting step, B0016-082BB was used as the wild type, and a plasmid harboring the Cg*panD* gene (Figure 1B) was transformed into B0016-082BB to enhance Cg*panD* expression, generating the B0016-01 strain. As shown in Figure 2A, with the reinforcement of the rate-limiting step, strain B0016-01 effectively produced β-alanine, with a concentration of 7.18 g/L, which was 10.72 times higher than that of the wild type, indicating that with the overexpression of Cg*panD*, the β-alanine production was enhanced.

### 3.3. Regulation between the β-Alanine Biosynthesis Module and the TCA Module

The yield of β-alanine is directly related to the stringency of the upstream pathway [23], and aspartase (AspA, encoded by *aspA*) from *E. coli* catalyzes the reaction from fumarate to L-aspartate (Figure 1A) [24]. Therefore, an increase in L-aspartate accumulation in vivo would enhance the supply of the precursor of β-alanine biosynthesis. To enhance the expression of *aspA*, the plasmid pETpL-Cg*panD*-*aspA* harboring Cg*panD* and *aspA* genes (Figure 1B) was constructed and transformed into the wild type; additionally, to release the induction of the lactose operon, the *lacI* gene was knocked out from both the strain and the plasmid, generating the B0016-02 strain. Compared with B0016-01, with the reinforcement of the reaction from fumarate to L-aspartate, β-alanine production increased by 15.59%, up to 8.30 g/L (Figure 2B). L-aspartate accumulated up to a concentration of 0.89 g/L at 68 h, then decreased rapidly to 0.19 g/L at 80 h. These results indicate that the accumulation of L-aspartate increased the production of β-alanine by acting as a precursor, which conforms with the previous report [25].

### 3.4. Regulation in the TCA Module

**Figure 3:**
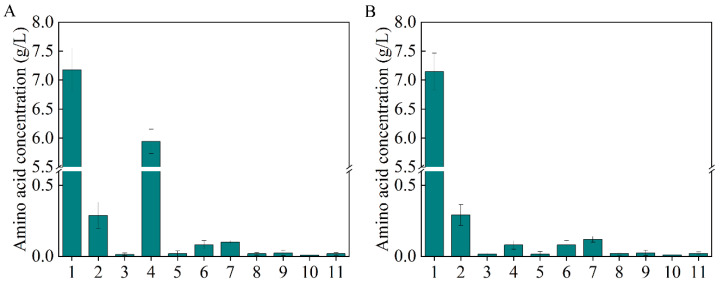
The total amino acids generated by B0016-01(**A**) and B0016-03 (**B**). 1: β-alanine; 2: L-aspartate; 3: glutamate; 4: valine; 5: methionine; 6: isoleucine; 7: leucine; 8: tyrosine; 9: phenylalanine; 10: γ-aminobutyric acid; 11: lysine.

As the most significant central carbon cycle, the TCA cycle plays an important role in energy and metabolite supply [26,27]. Moreover, it is directly related to L-aspartate and β-alanine production (Figure 1A). Metabolic regulation was carried out to channel the metabolic flux in the direction of L-aspartate and β-alanine generation. At the node of isocitrate, the metabolic flux from citrate has three different directions: malate (glyoxylate cycle), succinate (TCA cycle), and valine; the valine biosynthesis pathway is a branch of the TCA cycle (Figure 1A). The total amino acids were scanned to explore the competitive metabolic pathways, as shown in Figure 3A, and a large amount of valine was generated, which competes for metabolic flux with β-alanine biosynthesis. To keep the metabolic flux in the direction of β-alanine generation as far as possible, the valine generation pathway should be weakened or knocked out.

It was reported that the flow in the malate direction (glyoxylate cycle) is blocked due to the suppressed expression of *aceA*, *aceB*, and *acek* in glyoxylate shunt by *iclR*, a DNA-binding transcriptional repressor gene, and the glyoxylate shunt can be ignited with the knockdown of *iclR* (Figure 1A) [28]. Therefore, to weaken the valine biosynthesis pathway, *iclR* in B0016-02 was knocked out, generating the B0016-03 strain. Compared with B0016-02, though there were no significant effects on β-alanine production (8.10 g/L), cell density increased by 10.14% (Figure 2C), and the accumulated intracellular valine concentration was reduced to less than 0.1 g/L (Figure 3B), indicating that the deletion of *iclR* significantly reduced the generation of the byproduct valine, and the opening of the glyoxylate shunt enhanced cell growth.

The fumarate level is directly related to the downstream consumption pathway toward malate (Figure 1A). Though the reaction between fumarate and malate is reversible, this reaction works against fumarate accumulation. It was reported that the metabolic flux from fumarate to malate could be weakened with the knockdown of two fumarase genes (*fumA* and *fumC*) [24]; therefore, the *fumA* and *fumC* in B0016-03 were deleted (Figure 1A), generating the B0016-04 strain. Compared with that in B0016-03, the β-alanine production of B0016-04 increased by 10.49%, up to 8.95 g/L, and the biomass decreased by 21.49% (Figure 2D). These results demonstrate that the knockdown of *fumA* and *fumC* weakened the metabolic flux from fumarate to malate, resulting in the accumulation of fumarate and facilitating L-aspartate and β-alanine generation. However, the impaired metabolic flux to malate weakened the synthesis of oxaloacetate from malate. As a basic C4 metabolite, oxaloacetate is involved in many metabolic pathways, and an inadequate oxaloacetate level affects the basic intracellular metabolic processes, leading to a biomass decrease [29]. Therefore, increasing the biosynthesis of oxaloacetate based on metabolic flux regulation between the TCA module and the glycolysis module was subsequently carried out.

### 3.5. Regulation between the TCA Module and the Glycolysis Module

To reinforce the metabolic flux to oxaloacetate, the phosphoenolpyruvate carboxykinase (PPC, encoded by *ppc*) gene from *E. coli* was overexpressed using the plasmid pCDFPL-*ppc* (Figure 1B). The plasmid pCDFPL-*ppc* was transformed into B0016-04, generating the B0016-05 strain, which harbored the plasmids pETPL-Cg*panD*-*aspA* and pCDFPL-*ppc*. As shown in Figure 2E, compared with other strains, the cell density and β-alanine production decreased drastically and significantly, which indicated that an excessive expression of *ppc* may lead to a metabolic imbalance, thus inhibiting β-alanine generation and cellular growth. It is possible that the metabolic flux from pyruvate to oxaloacetate was strengthened by *ppc* overexpression; however, the metabolic flux at the pyruvate node remained constant. The pyruvate node is an important branching point in the metabolism of carbohydrates and is the key metabolic precursor of many second-generation metabolites [30]. The decreasing of pyruvate affected the whole balance of the metabolic pathway. Enhancing the metabolic flux to pyruvate might minimize the impact of *ppc* overexpression.

### 3.6. Regulation in the Glycolysis Module

Glycerol was used as the sole carbon source in this study; enhancement of the metabolization of glycerol could reinforce the flux to pyruvate (Figure 1A). It was reported that glycerol dehydrogenase (GldA, encoded by *gldA*) and PEP-dependent dihydroxyacetone kinase (DhaKLM, encoded by *dhaKLM*) are two key enzymes for glycerol metabolism [31]. The genes of *ppc*, *gldA*, and *dhaKLM* (Figure 1B) were constructed and transformed into B0016-04, generating the B0016-06 strain. Compared with that in B0016-04, cell density and β-alanine production increased by 3.13% and 6.59% (up to 9.54 g/L), respectively (Figure 2F), indicating that with the enhancement of glycerol utilization, the metabolic flux at the pyruvate node could be replenished efficiently, and then effectively distributed to the downstream pathways.

### 3.7. Intensive Regulation in the β-Alanine Biosynthesis Module

Various sources of L-aspartate-α-decarboxylase, such as *E. coli* [32], *C. glutamicum* [4,33], *Bacillus subtilis* [15], *Helicobacter pylori* [34], *Mycobacterium tuberculosis* [35], and *Tribolium castaneum* [34,35], have been reported, among which the L-aspartate-α-decarboxylase from *T. castaneum* (Tc*panD*) exhibited the highest activity [36,37]. As the rate-limiting step of β-alanine generation is the reaction from L-aspartate to β-alanine [17], to further increase β-alanine generation, the Cg*panD* used in B0016-06 was substituted by Tc*panD*, generating the B0016-07 strain, which harbored the plasmids pETpL-Tc*panD*-*aspA* and pCDFPL-*ppc*-*gldA*-*dhaKLM*. Compared with that in B0016-06, as shown in Figure 2G, β-alanine concentration was up to 10.47 g/L and less L-aspartate was accumulated in these strains. Compared with the wild type, β-alanine production of B0016-07 increased by more than 10 times.

### 3.8. Performance of the Microbial Cell Factory in Fed-Batch Fermentation

**Figure 4:**
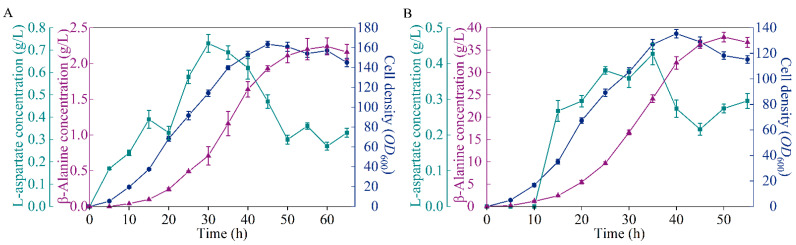
Comparison of the β-alanine production between B0016-07 (**B**) and the wildtype (**A**) in a 5 L bioreactor. Symbols: cell density, blue circles; β-alanine concentration, purple triangles; L-aspartate concentration, green squares.

To detect the performance of the microbial cell factory B0016-07, fed-batch fermentation was performed in 5 L bioreactors for comparison with the wild type. As shown in Figure 4, 37.93 g/L of β-alanine was generated by B0016-07 after 50 h incubation, which is 16.93 times higher than that of the wild type, but is 5.19 g/L less than the highest known yield reported [6]. By comparison, we speculate that β-alanine production could be further increased if metabolic flux was blocked at the nodes of L-aspartate and oxaloacetate to prevent the loss of metabolic flux in bypass pathways [6]. Further studies could be carried out for the continuous optimization of the metabolic pathway. The cell density of B0016-07 in the fermentation was lower than that of the wild type, which may be due to the inhibition of a high concentration of β-alanine, because product inhibition occurs when the β-alanine concentration is up to 32 g/L [16]. Another possibility is that β-alanine production competes for energy with cell growth, and enhancing the metabolic flux to β-alanine production resulted in less energy for cell growth. Regardless, high β-alanine production is the ultimate objective; the lower cell density, the higher the conversion.

## 4. Conclusions

The performance of microbial cell factories, including growth performance and production performance, is the key point to examine whether they can be used in industrial production. It is important for maintaining the balance between cell growth and production. In this study, the MME strategy was recruited for β-alanine generation by regulation throughout the entire β-alanine biosynthesis pathway, rationally and systematically. Only a slight metabolic burden existed while the metabolic flux was channeled in the direction of β-alanine generation. As a result, 37.93 g/L β-alanine was generated by B0016-07, which is the highest known yield. This study was meaningful to the synthetic biology of β-alanine industrial production.

## Figures and Tables

**Table 1:** Strains and plasmids used in this study.

Strains	Description	Source
B0016-082BB	B0016 Δ*ackA*-*pta* Δ*pflB* Δ*adhE* Δ*frdA* Δ*ldhA* Δ*lysC* Δ*panC* Δ*pts*, *panD*: Cg*panD*	[18]
B0016-01	B0016-082BB, pETpL-Cg*panD*	This study
B0016-02	B0016-01, Δ*lacI*, pETpL-Cg*panD*-*aspA*	This study
B0016-03	B0016-02, Δ*iclR*, pETpL-Cg*panD*-*aspA*	This study
B0016-04	B0016-03, Δ*fumA*, Δ*fumC*, pETpL-Cg*panD*-*aspA*	This study
B0016-05	B0016-04, pETpL-Cg*panD*-*aspA*, pCDFPL-*ppc*	This study
B0016-06	B0016-04, pETpL-Cg*panD*-*aspA*, pCDFPL-*ppc*-*gldA*-*dhaKLM*	This study
B0016-07	B0016-06, pETpL-Tc*panD*-*aspA*, pCDFPL-*ppc*-*gldA*-*dhaKLM*	This study

## Data Availability

The data presented in this study are available on request from the corresponding author.

## Supplementary Materials

The following are available online at https://www.mdpi.com/article/10.3390/biology10101017/s1, Table S1. Primers used in this study.
