# Supplementary material for: Enhancement of β-Alanine Biosynthesis in Escherichia coli Based on Multivariate Modular Metabolic Engineering
Source: Biology (Basel). 2021 Oct 9;10(10):1017. doi: 10.3390/biology10101017 (PMC8533518; doi:10.3390/biology10101017)
Supplement: Supplementary file 1 [file biology-10-01017-s001.zip › biology-1382320-supplementary.pdf]

Table S1. Primers used in this study.

Primers	Sequence (5'-3') restriction italic/underlined	Purpose
lacI-F1	TGAGCTAACTCACATTAATTGCGTTGCGCTCACTGCCCGCTTTCCAGTCGGTGTAGGCTGGAGCTGCTTC	Δ*lacI*
lacI-R1	GTATGGCATGATAGCGCCCGGAAGAGAGTCAATTCAGGGTGGTGAATGTGATTCCGGGGATCCGTCGACC	Δ*lacI*
iclR-F	AGAATATTGCCTCTGCCCGCCAGAAAAAGTCAGCGCATTCCACCGTACGCGTGTAGGCTGGAGCTGCTTC	Δ*iclR*
iclR-R	CAATAAAAATGAAAATGATTTCCACGATACAGAAAAAAGAGACTGTCATGATTCCGGGGATCCGTCGACC	Δ*iclR*
fumC-F	ATTCCACGGCTGCACCTGTATGTTGCAGATTAACGCCCGGCTTTCATACTGTGTAGGCTGGAGCTGCTTC	Δ*fumA*, Δ*fumC*
fumA-R	AACACCCGCCCAGAGCATAACCAAACCAGGCAGTAAGTGAGAGAACAATGATTCCGGGGATCCGTCGACC	Δ*fumA*, Δ*fumC*
lacI-F2	AGCGTCGAGATCCCGGATCTCGACCGATGCCCTTGA	pET24a, Δ*lacI*
lacI-R2	GCATCGGTCGAGATCCGGGATCTCGACGCTCTCC	pET24a, Δ*lacI*
p_L-F1	TGGTATTTATGTCAAATTTCGCGGGATCGAGATCTC	pET24a, T7::p_L
p_L-R1	TCGATCCCGCGAAATTTGACATAAATACCACTGGCGGTGATACTGAGCACATCACCTCTAGAAATAATTTTGTTTAACTTTAAGAAG	pET24a, T7::p_L

	GAG	
Cg*panD*-F1	CGCAGCATATGTATATCTCCTTCTTAAAG TTAAACAAAATTATTTCTAGAGG	pET24a-Cg*panD*
Cg*panD*-R1	AGCCGCAGCATCTAATGAGATCCGGCTG CTAACAAAG	pET24a-Cg*panD*
Cg*panD*-F2	ACTTTAAGAAGGAGATATACATATGCTGC GCACCATTCTGG	pET24a-Cg*panD*
Cg*panD*-R2	AGCAGCCGGATCTCATTAGATGCTGCGGC TGGTCAG	pET24a-Cg*panD*
aspA-F1	TTTGACATATGTATATCTCCTTCTTAAAGT TAAACAAAATTATTTCTAGAGG	pET24a-*aspA*
aspA-R1	GAAAGCGAACAGTAATGAGATCCGGCTG CTAACAAAG	pET24a-*aspA*
aspA-F2	ACTTTAAGAAGGAGATATACATATGTCA AACAACATTCGTATCGAAGA	pET24a-*aspA*
aspA-R2	AGCAGCCGGATCTCATTACTGTTCGCTTT CATCAGTATAGCG	pET24a-*aspA*
Cg*panD*-*aspA*-F1	CGATCCTCTACGCCGGTGGGTATGGTGGC AGGCC	pET24a-Cg*panD*-*aspA*
Cg*panD*-*aspA*-R1	AACTATATCCGGATTGCCGAAACAAGCG CTCATGAG	pET24a-Cg*panD*-*aspA*
Cg*panD*-*aspA*-F2	TGCCACCATACCCACCGGCGTAGAGGAT CGAGATCT	pET24a-Cg*panD*-*aspA*
Cg*panD*-*aspA*-R2	AGCGCTTGTTTCGGCAATCCGGATATAGT TCCTCCTTTCAG	pET24a-Cg*panD*-*aspA*
Tc*panD*-F1	GCCGGCATATGTATATCTCCTTCTTAAAG TTAAACAAAATTATTTCTAGAGG	pET24a-Tc*panD*
Tc*panD*-R1	GGTTCTGACCTGTAATGAGATCCGGCTGC TAACAAAG	pET24a-Tc*panD*

Tc*panD*-F2	ACTTTAAGAAGGAGATATACATATGCCG GCTACCGGTGAAGAC	pET24a-Tc*panD*
Tc*panD*-R2	AGCAGCCGGATCTCATTACAGGTCAGAA CCCAGACGTTCG	pET24a-Tc*panD*
Tc*panD*-*aspA*-F1	CGATCCTCTACGCCGGTGGGTATGGTGGC AGGCC	pET24a-Tc*panD-aspA*
Tc*panD*-*aspA*-R1	AACTATATCCGGATTGCCGAAACAAGCG CTCATGAG	pET24a-Tc*panD-aspA*
Tc*panD*-*aspA*-F2	TGCCACCATACCCACCGGCGTAGAGGAT CGAGATCT	pET24a-Tc*panD-aspA*
Tc*panD*-*aspA*-R2	AGCGCTTGTTTCGGCAATCCGGATATAGT TCCTCCTTTCAG	pET24a-Tc*panD-aspA*
ppc-F1	TCGTTCATATGTATATCTCCTTCTTAAAGT TAAACAAAATTATTTCTAGAGG	pET24a-*ppc*
ppc-R1	CGTAATACCGGCTAATGAGATCCGGCTGC TAACAAAG	pET24a-*ppc*
ppc-F2	ACTTTAAGAAGGAGATATACATATGAAC GAACAATATTCCGCATT	pET24a-*ppc*
ppc-R2	AGCAGCCGGATCTCATTAGCCGGTATTAC GCATACCTG	pET24a-*ppc*
gldA-F1	CGGTCCATATGTATA TCTCCTTCTTAAAGTTAAACAAAATTATT TCTAGAGG	pET24a-*gldA*
gldA-R1	CAAGAGTGGGAATAATGAGATCCGGCTG CTAACAAAG	pET24a-*gldA*
gldA-F2	ACTTTAAGAAGGAGATATACATATGGAC CGCATTATTCAATCACC	pET24a-*gldA*
gldA-R2	AGCAGCCGGATCTCATTATTCCCACTCTT GCAGGAAACG	pET24a-*gldA*

dhaKLM-F1	TTTTTCATATGTATATCTCCTTCTTAAAGT TAAACAAAATTATTTCTAGAGG	pET24a-*dhaKLM*
dhaKLM-R1	AACCGTCAGGGTTAATGAGATCCGGCTG CTAACAAAG	pET24a-*dhaKLM*
dhaKLM-F2	ACTTTAAGAAGGAGATATACATATGAAA AAATTGATCAATGATGTGCA	pET24a-*dhaKLM*
dhaKLM-R2	AGCAGCCGGATCTCATTAACCCTGACGGT TGAAACGTT	pET24a-*dhaKLM*
ampr-F1	ATTAAGCATTGGTAATGTCTAACAATTCG TTCAAGCCG	pCDF, *ampr*
ampr-R1	ATGTTGAATACTCATACTCTTCCTTTTTCA ATATTATTGAAGCATTT	pCDF, *ampr*
ampr-F2	ACGAATTGTTAGACATTACCAATGCTTAA TCAGTGAGGC	pCDF, *ampr*
ampr-R2	TGAAAAAGGAAGAGTATGAGTATTCAAC ATTTCCGTGTCG	pCDF, *ampr*
lacI-F3	AGAGCGTCGAGATCCCAGTGAGCGCAAC GCAATTAAT	pCDF, Δ*lacI*
lacI-R3	GCGTTGCGCTCACTGGGATCTCGACGCTC TCCCTTATG	pCDF, Δ*lacI*
ppc-F3	TGGTATTTATGTCAATCACTCATTAGGCA CCGGGA	pCDF-*ppc*
ppc-R3	TTGAGGGGTTTTTTGGCTAACTTACATTA ATTGCGTTGCG	pCDF-*ppc*
ppc-F4	GTGCCTAATGAGTGATTGACATAAATACC ACTGGCGGT	pCDF-*ppc*
ppc-R4	TTAATGTAAGTTAGCCAAAAAACCCCTCA AGACCCG	pCDF-*ppc*
gldA-F3	TGGTATTTATGTCAATCACTCATTAGGCA	pCDF-*gldA*

	CCGGGA	
gldA-R3	TTGAGGGGTTTTTGGCTAACTTACATTA ATTGCGTTGCG	pCDF-*gldA*
gldA-F4	GTGCCTAATGAGTGATTGACATAAATACC ACTGGCGGT	pCDF-*gldA*
gldA-R4	TTAATGTAAGTTAGCCAAAAACCCCTCA AGACCCG	pCDF-*gldA*
ppc-*gldA*-*dhaKLM*-F1	TGGTATTTATGTCAACCAGTGAGCGCAAC GCATAAG	pCDF-*ppc*-*gldA*-*dhaKLM*
ppc-*gldA*-*dhaKLM*-R1	TTCAACCGTCAGGGTTAATTAACCTAGGC TGCTGCCACC	pCDF-*ppc*-*gldA*-*dhaKLM*
ppc-*gldA*-*dhaKLM*-F2	CGTTGCGCTCACTGGTTGACATAAATACC ACTGGCG	pCDF-*ppc*-*gldA*-*dhaKLM*
ppc-*gldA*-*dhaKLM*-R2	ATCGCCGACATCACCGGATATAGTTCCTC CTTTCAGC	pCDF-*ppc*-*gldA*-*dhaKLM*
ppc-*gldA*-*dhaKLM*-F3	GGAGGAACTATATCCGGTGATGTCGGCG ATATAGGC	pCDF-*ppc*-*gldA*-*dhaKLM*
ppc-*gldA*-*dhaKLM*-R3	AGCCTAGGTTAATTAACCCTGACGGTTGA AACGTTG	pCDF-*ppc*-*gldA*-*dhaKLM*
